# Analysis of lumbar lateral instability on upright left and right bending radiographs in symptomatic patients with degenerative lumbar spondylolisthesis

**DOI:** 10.1186/s12891-022-05017-1

**Published:** 2022-01-17

**Authors:** Xin-wen Wang, Xi Chen, Yang Fu, Xiao Chen, Feng Zhang, Hai-ping Cai, Chang Ge, Wen-zhi Zhang

**Affiliations:** Department of Orthopedics, Spine Center, The First Affiliated Hospital of USTC: Anhui Provincial Hospital, No. 17, Lujiang Road, Hefei, 230001 China

**Keywords:** Lumbar lateral instability, Disc wedging, Degenerative lumbar spondylolisthesis, Low back pain

## Abstract

**Background:**

To evaluate lumbar mobility in various positions using upright left and right bending radiographs in patients with degenerative lumbar spondylolisthesis (DLS), as well as to assess the impact of lateral instability on patient-reported outcomes.

**Methods:**

This study retrospectively reviewed a consecutive series of patients with DLS between January 2019 and October 2020. The enrolled patients were divided into two groups: the lateral instability group (group L) and non-lateral instability group (group NL). Translational and angular motion in both sagittal and coronal planes and patient-reported outcomes were compared between the two groups.

**Results:**

There were 104 (59.8%) patients in group L and 70 (40.2%) patients in group NL, with an average age of 60.6 ± 7.8 years. Patients with a right bending posture in group L had a higher slip percentage (14.2 ± 7.4% vs 9.2 ± 3.2%, *p* = 0.01) and slip angle (6.3 ± 1.5° vs 2.2 ± 0.8°, *p* = 0.021). Compared with group NL, group L demonstrated significantly larger angular motion in the coronal plane (2.4 ± 1.3° vs 1.0 ± 0.7°, *p* = 0.008). Patients with lateral instability had worse preoperative back pain (6.1 ± 1.6 vs 2.7 ± 1.9, *p* = 0.01) and Oswestry Disability Index (ODI) scores (37.7 ± 5.5 vs 25.6 ± 2.6, *p* = 0.002). In terms of pain characteristics, group L was characterized by pain when getting out of a car, when rising from a chair, and when climbing stairs (all *p* values < 0.05).

**Conclusion:**

Lumbar lateral instability, that is, increased mobility in the coronal plane on lateral bending radiographs, translational and/or angular, correlates to more pronounced patient related symptoms in degenerative L4–5 spondylolisthesis. The existence of lumbar lateral instability leads to worse impacts on patient-reported outcomes when patients change their positions including getting out of a car, rising from a chair, and climbing stairs.

## Background

Degenerative lumbar spondylolisthesis (DLS) is a common pathological condition defined by anterior slippage of the upper vertebra in relation to lower vertebra occurring in the involved segment with intact neural arch, that appears frequently in the aging population at the L4/5 level [1, 2]. Nearly 22% of symptomatic patients seek surgical treatment [3, 4]. And surgical methods are diverse, including decompression alone or spinal decompression and fusion surgery, with or without interbody fusion [5]. Lumbar stability is the most important consideration when evaluating DLS. There are many methods to assess lumbar stability, including flexion-extension radiographs; computed tomography (CT), assessing facet joint effusion; and magnetic resonance imaging (MRI), evaluating disc degeneration [6–8]. However, it is worth noting that these theoretical foundations and evaluation methods concerning lumbar instability are absolutely based on the sagittal plane. For example, the definition of lumbar instability was simply determined as sagittal translation > 3 mm and/or angulation > 8° [9].

The lumbar vertebrae function as a whole spinal unit, and they display a range of vertebral motions in both sagittal and coronal planes [10]. In our clinical practice, we noticed that nearly 60% of patients with DLS had lateral displacement and apparent disc wedging at the same slipped level, suggesting significant displacement beyond the normal range between the lower and upper lumbar vertebrae in the coronal plane. As revealed in a study using kinematic CT, increases in disc wedging correlate with increased rotational hypermobility of the L4/5 disc, suggesting that there is abnormal lumbar instability in the coronal plane in patients with an extremely rotational position [11]. However, unlike abundant studies on lumbar instability in the sagittal plane, lateral instability in the coronal plane has rarely been reported. Although the mechanism of lumbar lateral displacement remains unclear, further studies concerning lateral instability in the coronal plane in a distinct subgroup of patients with DLS will be helpful to define lumbar instability.

We hypothesized that patients with lateral displacement and/or disc wedging had lumbar lateral instability. Thus, the main purpose of this study is to evaluate lumbar mobility in various positions using upright left and right bending radiographs, as well as to assess the impacts of lateral instability on patient-reported outcomes.

## Methods

### Patients

This project was designed as an observational retrospective study. With institutional review board approval obtained, 174 patients who were diagnosed with DLS in our hospital during January 2019 and October 2020 were reviewed retrospectively. Patients enrolled in this study had to meet the following criteria, including above 50 years of age, with low back pain, one segment L4/5 DLS only, and a complete set of radiological data with upright left and right bending (LRB) radiographs and flexion and extension (FE) radiographs. Patients meeting any of the following criteria, including spondylolisthesis at any other level in the lumbar spine, unequal length of lower limbs and prior history of spinal trauma, scoliosis, tumor, surgery or infection, were excluded. Patient demographic and clinical data, such as age, sex, BMI, height, weight, occupation and marital status, were collected based on medical records.

### Patient-reported outcome measurement

Patient-reported outcomes (PROs) were evaluated using visual analog scale (VAS) for back pain, VAS for leg pain, and Oswestry disability index (ODI) scale [12, 13]. In addition, pain features were evaluated through questionnaires completed by the patients, which consisted of the following items: pain on rising from a chair, pain on prolonged standing, pain on walking some distance, pain on getting out of a car and pain on climbing stairs.

### Radiographic evaluation

All the flexion and extension radiographs measurements in the standing position (FE) and upright left and right bending (LRB) radiographs measurements were obtained by 3 independent reviewers and an average of their readings was recorded. It is worth mentioning that patients adopt standard lateral bending postures when taking upright left and right bending radiographs, which were similar to the postures of some patients with scoliosis when taking left and right bending radiographs to evaluate the spinal flexibility clinically. To take the left bending radiograph, the patient’s left hand was tightly pressed against the waist, and the right upper arm was raised over the top of the head to the left; similarly, when taking the right bending radiograph, the patient’s right hand was tightly pressed against the waist and the left arm was raised above the head to the right. Slip distance was measured as the interval between two extended lines of the posterior aspects of the L4 and L5 vertebral bodies on FE radiographs, while it was measured between two extended lines of the left or right side of the L4 and L5 vertebral bodies on upright LRB radiographs [14]. The slip percentage (SP) was measured as the ratio of the slip distance of L4 to the width of the L5 vertebral upper endplate. The slip angle (SA) was measured as the difference in the angles between lower endplate of the L4 vertebra and the upper endplate of the L5 vertebra in the coronal and sagittal planes.

On neutral upright radiographs, anterior disc height (ADH), the posterior disc height (PDH), left disc height (LDH) and right disc height (RDH) were measured. The ADH was measured as the vertical distance between the lower anterior conner of L4 and upper endplate of L5 in the sagittal plane, whereas the PDH was measured as the same method. The LDH was measured as the vertical distance between the leftmost point of L4 and upper endplate of L5 in the coronal plane, while the RDH was measured as the same method. In order to control measured bias in radiographs, we calculated the ratio of the disc height to the length of the upper endplate of L5. The angle of lumbar lordosis (LL) was measured as the difference in the angles between the upper L1 endplate and upper S1 endplate in the sagittal plane.

The sagittal translation distance of L5 vertebra from F to E and the lateral translation distance of L5 vertebra from LB to RB were measured as translational motion. The percentage of the translation distance to L4 vertebra was used for the final analysis. Angular motion was also measured as the difference in the intervertebral angles at the L4/5 level from E to F or LB to RB.

### Study groups and statistical analysis

The patients meeting the inclusion criteria were divided into two groups, the lateral instability group (group L) and the non-lateral instability group (group NL), based on the slip parameters on FE and upright LRB radiographs. Besides, a lateral translation > 5 mm and/or lateral disc wedging > 5° was identified as lateral instability [15–17]. Group L included patients with a definite lateral translation > 5 mm and/or lateral disc wedging > 5° of the left or right side on lumbar vertebral bodies. Group NL included patients with no lateral instability.

All data were analyzed using SPSS version 17.0 (SPSS; Chicago, IL, USA) and were presented as mean ± standard deviation. Independent sample t-tests were used to analyze the differences in terms of patient demographic and clinical data between group L and group NL, while paired t-tests were used to analyze the intra-group differences on FE and upright LRB radiographs to evaluate instability. A *p* value < 0.05 was considered statistically significant.

## Results

### Patient’s demographic and clinical data analysis

A total of 174 patients (36 males and 138 females) were included in this study, with an average age of 60.6 ± 7.8 years, ranging between 55 and 77 years. Patients were divided into groups, with or without lateral instability, and then compared. One hundred and four (59.8%) patients were assigned to group L, and 70 (40.2%) patients were assigned to group NL. No statistically significant difference was observed in sex, age, height, weight, BMI, occupation or marital status between group L and group NL, as shown in Table 1.

**Table 1:** Patient’s demographic and clinical data analysis

Variable/Groups	group L (*n* = 104)	group NL (*n* = 70)	*p*
Gender			
Male	20 (19.2%)	16 (22.8%)	0.67
Female	84 (80.8%)	54 (77.2)	
Age (year)	61.7 ± 7.4	59.4 ± 8.3	0.46
Weight (kg)	62.4 ± 10.7	62.4 ± 10.7	0.23
Height (cm)	160.1 ± 7.7	158.4 ± 5.8	0.19
BMI (kg/m^2)	24.4 ± 3.7	24.6 ± 3.4	0.63
Occupation			
Housewife	48 (46.2%)	16 (22.8%)	0.45
Sedentary	26 (25.0%)	14 (20.0%)	
Physically tiring work	16 (15.4%)	30 (42.8%)	
Retired	14 (13.4%)	10 (14.4%)	
Marital status			
Single	10 (9.6%)	8 (11.4%)	0.32
Married	94 (90.4%)	62 (88.6%)	

* Statistically significant between the group L and group NL

### Comparison of slip parameters between group L and group NL

**Fig. 1:**
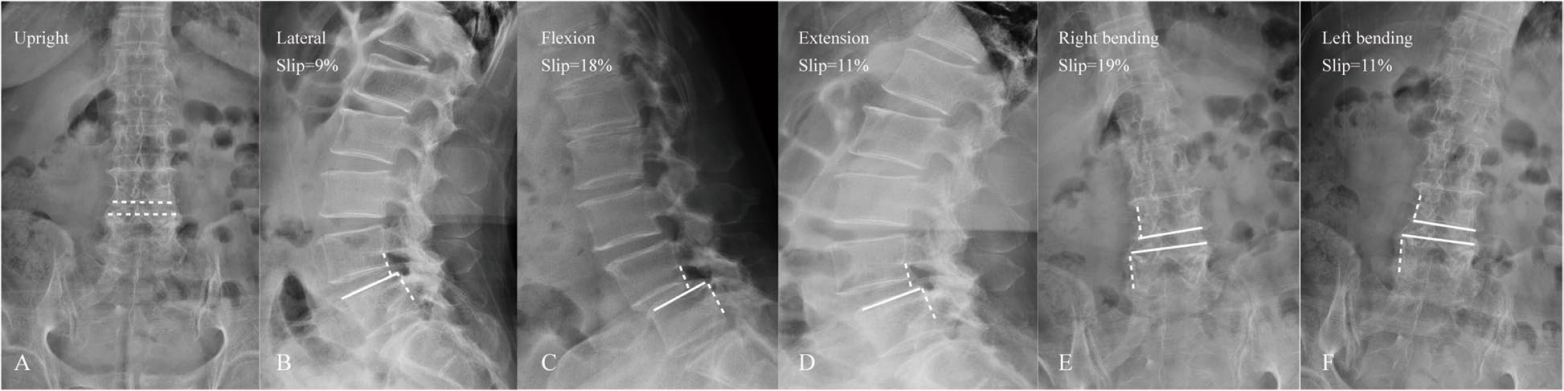
A 65-year-old female with degenerative lumbar spondylolisthesis. The upright neutral (**A**) and lateral (**B**) radiograph showing a slipped level at the L4/5 segment. The flexion (**C**) and extension (**D**) radiograph showing lumbar instability on the sagittal plane. The group L with lateral instability demonstrated a significantly high slip percentage on left and right bending radiographs (**E**, **F**)

**Fig. 2:**
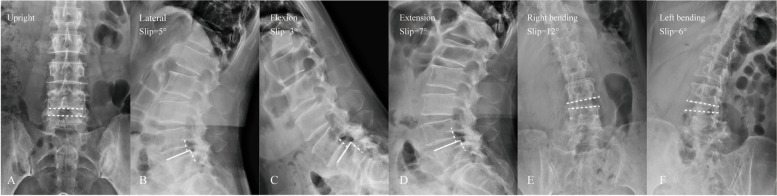
A 58-year-old female with degenerative lumbar spondylolisthesis. The upright neutral (**A**) and lateral (**B**) radiograph showing a slipped level at the L4/5 segment. The flexion (**C**) and extension (**D**) radiograph showing lumbar instability on the sagittal plane. The group L with lateral instability demonstrated significantly high slip angle on left and right bending radiographs and large angular motion on the coronal plane (**E**, **F**) (2.4 ± 1.3° vs 1.0 ± 0.7°, *p* = 0.008)

The measurements of slip parameters are summarized in Table 2. Compared with group NL, group L with a right bending posture demonstrated a significantly higher slip percentage (14.2 ± 7.4% vs 9.2 ± 3.2%, *p* = 0.01) **(**Fig. 1**)**. Group L with a left bending posture had a higher slip angle than group NL (4.9 ± 1.9° vs 3.1 ± 1.0°, *p* = 0.01). Besides, the slip angle in group L with a right bending posture was significantly higher than that in group NL (6.3 ± 1.5° vs 2.2 ± 0.8°, *p* = 0.021) **(**Fig. 2**)**. The lumbar lordosis angle (LL) in group L was significantly lower than that in group NL (2.3 ± 0.7° vs 4.2 ± 0.5°, *p* = 0.034).

**Table 2:** Comparison of slip parameters between group L and group NL

	group L	group NL	*p*
Translation in the sagittal plane (%)	(SP)		
Flexion	20.3 ± 11.8	18.3 ± 10.1	0.862
Extension	19.5 ± 11.1	14.8 ± 8.3	0.217
Translation in the coronal plane (%)	(SP)		
Left bending	12.8 ± 6.4	9.9 ± 4.1	0.148
Right bending	14.2 ± 7.4	9.2 ± 3.2	0.01*
Angulation in the sagittal plane (°)	(SA)		
Flexion	3.9 ± 1.4	4.8 ± 2.4	0.005*
Extension	6.1 ± 2.5	5.2 ± 1.5	0.046*
Angulation in the coronal plane (°)	(SA)		
Left bending	4.9 ± 1.9	3.1 ± 1.0	0.01*
Right bending	6.3 ± 1.5	2.2 ± 0.8	0.021 *
Disc height on neutral radiographs	(mm)		
ADH	9.7 ± 1.1	9.4 ± 1.1	0.952
PDH	9.1 ± 0.9	9.2 ± 0.7	0.825
LDH	8.7 ± 0.7	8.4 ± 0.7	0.921
RDH	9.6 ± 0.9	9.3 ± 0.7	0.357
Angle of Lumbar lordosis (LL) (°)	2.3 ± 0.7	4.2 ± 0.5	0.034*

### Comparison of translational motion and angular motion between group L and group NL

As shown in Table 3, translational motion in the sagittal plane was significantly higher in group L than in group NL (5.9 ± 2.8% vs 4.6 ± 4.1%, *p* = 0.003). Compared with group NL, group L demonstrated significantly larger angular motion in the coronal plane (2.4 ± 1.3° vs 1.0 ± 0.7°, *p* = 0.008) **(**Fig. 2**)**.

**Table 3:** Comparison of translational motion and angular motion between group L and group NL

	group L	group NL	*p*
Translational motion in sagittal plane (%)	5.9 ± 2.8	4.6 ± 4.1	0.003*
Translational motion in coronal plane (%)	5.9 ± 5.4	3.2 ± 2.8	0.373
Angular motion in sagittal plane (°)	2.5 ± 1.8	2.1 ± 1.1	0.11
Angular motion in coronal plane (°)	2.4 ± 1.3	1.0 ± 0.7	0.008*

* Statistically significant between the group L and group NL

### Evaluation of PROs and pain characteristics

The PROs and pain characteristic evaluations are summarized in Table 4. Group L had worse preoperative back pain (6.1 ± 1.6 vs 2.7 ± 1.9, *p* = 0.01) and ODI scores (37.7 ± 5.5 vs 25.6 ± 2.6, *p* = 0.002) than Group NL, but no difference in the VAS score for leg pain was detected. In terms of pain characteristics, group L with lateral instability was characterized by pain when getting out of a car, when rising from a chair, and when climbing stairs (all *p* values < 0.05).

**Table 4:** Patient-reported outcomes and pain characters evaluations

Variable/Groups	group L	group NL	*p*
VAS (Back)	6.1 ± 1.6	2.7 ± 1.9	0.010*
VAS (Leg)	5.5 ± 0.9	1.8 ± 0.7	0.232
ODI	37.7 ± 5.5	25.6 ± 2.6	0.002*
Pain on rising from a chair			
Yes	90 (86.5%)	18 (25.7%)	0.023*
No	14 (13.5%)	52 (74.3%)	
Pain on prolong standing			
Yes	82 (78.8%)	28 (40.0%)	0.064
No	22 (21.2%)	42 (60.0%)	
Pain on walking some distance			
Yes	76 (73.1%)	48 (68.6%)	0.78
No	28 (26.9%)	22 (31.4%)	
Pain on getting out of a car			
Yes	76 (73.1%)	24 (34.3%)	0.012*
No	28 (26.9%)	46 (65.7%)	
Pain on climbing stairs			
Yes	72 (69.2%)	34 (48.6%)	0.026*
No	32 (30.8%)	36 (51.4%)	

VAS indicates visual analogue scale; ODI indicates oswestry disability index

* Statistically significant between the group L and group NL

## Discussion

Currently, DLS is one of the most common diagnoses prompting the decision of surgical intervention in patients with low back pain [18]. Many patients choose surgical treatment mainly due to serious back pain and failure of conservative treatments. Theoretically, the causes of low back pain may be multifactorial, including chronic injury, inflammation, lumbar disc degeneration and fatty acids of paraspinal muscles, but the primary causes are related to lumbar instability in DLS [19–22]. In a recent study, Iguchi T et al. [23]. conducted a survey by using a large number of 477 age-matched patients, and the results demonstrated that forward translation of the involved segments had a greater influence on symptomatic pain and that the presence of dynamic instability could be an indicator for persistent low back pain. However, our findings are not exactly consistent with the study mentioned above to some extent. Some patients with pain did not show obvious segmental instability on FE radiography. Therefore, lumbar FE radiography is ineffective in detecting lumbar instability in some special populations, or there are no signs of lumbar segmental instability in patients with occasional low back pain.

DLS is a heterogeneous disease that displays a large variation in clinical and imaging manifestations. Additionally, the features of low back pain are very different. As in our results, some patients complained of back pain when they were getting up from seats or bending forward to pick up things, while the others demonstrated severe pain when changing postures from an upright standing position to a left or right bending position. This revealed that some factors, not only in the lumbar sagittal plane but also in the coronal plane, resulted in back pain. In a study, Takahashi T et al. [16]. revealed that some patients with lateral vertebral translation in the lumbar local coronal plane had more severe pain. However, the article did not identify the relationship between lumbar coronal factors and low back pain.

The factors originating from the lumbar coronal plane can also be involved in the mechanism of low back pain. Theoretically, the progression of DLS can be classified into three distinct stages: the dysfunction stage, unstable stage, and stable stage. In the unstable stage, the lumbar vertebrae can not only move back and forth in the sagittal plane but also move left and right in the coronal plane. Previously, Iguchi et al. [24]. revealed that disc height showed an intimate relationship with lumbar instability, especially at the L4/5 segment, and apparent anterior translation should be considered evidence of lumbar segmental instability by FE radiography. On the other hand, loss of segmental disc space usually occurred at the same disc level as segmental wedging, and DLS could be followed by assessment of lumbar instability in the coronal plane [25]. However, large numbers of studies have mainly reported the relationship between low back pain and lumbar sagittal instability, and knowledge concerning lateral translation and lumbar instability in the coronal plane has often been ignored.

Local coronal instability (LCI) should be considered a sign of lumbar spine instability. As in our results, as many as 57% of patients with LCI demonstrated lateral translation > 5 mm or lateral disc wedging > 5° in the coronal plane, and were divided into Group L, which demonstrated higher lumbar local coronal motion and segmental instability than group NL. Theoretically, the intervertebral disc is an important structure that supports lumbar spine stability, and the ability of the disc and ligament complex to sustain shear forces of lumbar vertebrae highly depends on the tensile strain of the inner fibrous ring and ligaments, which result from the expansion pressure of the disc. When the disc collapses, the lumbar coronal or sagittal motion increases. Asymmetric degeneration of the intervertebral disc causes changes in the biomechanics of the lumbar spine. The range of coronal motion on the right side, consistent with our study, was higher than that on the left side. This theoretical background can be supported by a previous study concerning degenerative scoliosis, in which an asymmetrical collapse of the disc and asymmetrical incompetence and hypertrophy of the facet joints could cause lateral and rotational deformities, leading to degenerative lumbar coronal instability. Additionally, another study also suggested that patients with higher unilateral disc narrowing or longer vertebral osteophytes on one side had an increased incidence of lumbar coronal instability in the following decade [26].

In our study, patients with LCI had a greatly lower lumbar lordosis angle than DLS patients without LCI. A similar finding was observed in the early phase of degenerative lumbar scoliosis. James W et al. [27] noted that 171 out of 200 patients with degenerative scoliosis had less lordosis than normal subjects. In addition, in a current study, Yasuaki et al. [28] reported that of the 79 discs that showed a larger decrease in segmental lordotic wedging, progression of scoliotic wedging at the same level was observed in 56 cases. With initial wedging, the remaining vertebrae consequentially wedge toward the opposite apex to maintain balance and eventually trigger the loss of intervertebral disc space, resulting in LCI after a certain amount of time. Theoretically, degenerative scoliosis often occurs with DLS, and the risk factors are similar. Thus, lumbar scoliosis and LCI interact and influence each other.

DLS patients with LCI suffered from more severe low back pain. Although the instability of the sagittal plane was considered an important factor that was involved, the coronal plane factor also played a nonnegligible role in triggering pain, especially when rising from a chair or getting out of a car. Our findings revealed the possible reasons why coronal movements aggravated pain. Thus, LCI should be considered a factor when there is no obvious lumbar sagittal instability but with symptomatic low back pain triggered by getting out of a car or rising from a chair.

The main limitation of this study is the small sample size, as an adequate sample size would be more convincing. In addition, due to the lack of criteria, the radiological definition of LCI is not uniform and remains controversial. Furthermore, the use of only X-ray is inefficient for detecting some other manifestations of lumbar instability, including disc degeneration, facet joint hypertrophy and osteophyte formation. The presence of lumbar foraminal stenosis cannot be fully assessed owing to the lack of MRI scans, which affects the preoperative pain score largely. Additionally, the clinical magnification error in lateral plane radiographs should be considered in our study, which is closely related to BMI [29, 30]. There is a limitation in regard to clinical decision-making without the comparison to morphometric data from CT or MRI. Thus, further investigations should be conducted to clarify the relationship between low back pain and lumbar instability.

## Conclusion

Lumbar lateral instability, that is, increased mobility in the coronal plane on lateral bending radiographs, translational and/or angular, correlates to more pronounced patient related symptoms in degenerative L4–5 spondylolisthesis. The existence of lumbar lateral instability leads to worse impacts on patient-reported outcomes when patients change their positions including getting out of a car, rising from a chair, and climbing stairs.

## Data Availability

The analyzed data sets generated during the current study are available from the corresponding author on reasonable request.

## Abbreviations

DLS: Degenerative lumbar spondylolisthesis
L: Lateral
NL: Non-lateral
CT: Computed Tomography
MRI: Magnetic Resonance Imaging
LRB: Left and right bending
FE: Flexion and extension
ODI: Oswestry disability index
VAS: Visual analog scale
PROs: Patient-reported outcomes
SP: Slip percentage
SA: Slip angle
ADH: Anterior disc height
PDH: Posterior disc height
LDH: Left disc height
RDH: Right disc height
LL: Lumbar lordosis
LB: Left bending
RB: Right bending
E: Extension
F: Flexion
LCI: Local coronal instability
L4: Lumbar 4
L5: Lumbar 5
L4/5: Lumbar 4/5
